# Accuracy of self-collected versus healthcare worker collected specimens for diagnosing sexually transmitted infections in females: an updated systematic review and meta-analysis

**DOI:** 10.1038/s41598-024-61358-y

**Published:** 2024-05-07

**Authors:** Ziningi Nobuhle Jaya, Witness Mapanga, Thobeka Dlangalala, Nokukhanya Thembane, Kabelo Kgarosi, Tafadzwa Dzinamarira, Tivani Phosa Mashamba-Thompson

**Affiliations:** 1 School of Health Systems and Public Health, Faculty of Health Sciences, University of Pretoria, Pretoria, South Africa; 2 Department of Biomedical Science, Faculty of Natural Science, Mangosuthu University of Technology, Umlazi, KwaZulu-Natal South Africa; 3 Department of Library Services, Faculty of Health Sciences, University of Pretoria, Pretoria, South Africa; 4 Faculty of Health Sciences, University of Pretoria, Pretoria, South Africa

**Keywords:** Self-collect, Sexually transmitted infections, Diagnostic specimens, Females, Women, Diseases, Health care

## Abstract

The use of self-collected specimens as an alternative to healthcare worker-collected specimens for diagnostic testing has gained increasing attention in recent years. This systematic review aimed to assess the diagnostic accuracy of self-collected specimens compared to healthcare worker-collected specimens across different sexually transmitted infections (STIs) including *Chlamydia trachomatis* (CT), human papillomavirus (HPV), *Mycoplasma genitalium* (MG), *Neisseria gonorrhoea* (NG), *Treponema pallidum* and *Trichomonas vaginalis* (TV) in females. A rigorous process was followed to screen for studies in various electronic databases. The quality of included studies was assessed using the Quality Assessment of Diagnostic Accuracy Studies 2 tool. There were no studies on syphilis that met the criteria for inclusion in the review. A total of six studies for chlamydia, five studies for HPV, four studies for MG, and seven studies for gonorrhoea and trichomoniasis were included in the review. However, not all studies were included in the sub-group meta-analysis. The analysis revealed that self-collected specimens demonstrated comparable diagnostic accuracy to healthcare worker-collected specimens across most STIs. This indicates that the diagnostic accuracy of self-collected specimens can provide accurate results and enhance access to diagnostic testing, potentially improving healthcare service delivery. Future research should further explore the diagnostic accuracy of self-collected specimens in larger and more diverse populations.

## Introduction

Sexually transmitted infections (STIs) are a major global health concern that causes symptomatic and asymptomatic infections^1,2^. Most STIs caused by bacteria and parasites are curable if diagnosed and treated accordingly but all viral STIs are incurable^3,4^. The largest portion of STIs occurs in females across the globe^2,5^. In females, the consequence of undiagnosed and untreated STIs can result in reproductive health complications that include infertility, stillbirths, cancer development and increased susceptibility to HIV^1,2,4,6,7^. Considering this, our study focused on STIs in females.

Governments across the globe, particularly in low-and-middle-income countries (LMICs) continue to use syndromic management of STIs due to a scarcity of resources and restricted access to diagnostic laboratories^8,9^. This approach relies on reported signs and symptoms, and physical examinations for diagnosis and then treatment is issued for the most common STIs^8,10^. In light of this, it deters infected individuals who fear invasive genital examinations and stigmatization associated with STIs^11^. Additionally, this approach cannot address asymptomatic infections because these individuals may not seek care^8,12^. As such, asymptomatic individuals continue to spread infection and become susceptible to long term STI complications. Syndromic management often promotes over-diagnosing and over-treating because treatment is issued often targeting the most common STI causative pathogens instead of a specific pathogen^13,14^. As such Murewanhena et al.^14^ suggest a shift from syndromic management of STIs to a more pathogen specific diagnosis and treatment of STIs. The development of innovative alternative interventions, such as self-sampling, is key to improving STI healthcare service provision^15–17^. Self-sampling enables individuals to self-collect specimens for STI diagnosis either at home or healthcare facilities, providing convenience and accessibility in testing^18^. This intervention can be used to screen for asymptomatic infections^11^, and screen infections in remote areas where access to quality healthcare is limited^19,20^. Based on this, self-sampling can address the challenges linked to the syndromic management of STIs^19,21^. However, self-sampling may jeopardise specimen quality since the collection is performed by inexperienced individuals.

Since the potential of self-sampling interventions for STI diagnosis is evident, it is imperative to determine their diagnostic accuracy and reliability. A scoping review conducted by Jaya et al.^22^ in 2021 presented evidence that supports self-sampling interventions as appropriate alternatives to physician collected specimens for STI diagnosis. A meta-analysis conducted in 2005 proved that self-collected swabs were suitable alternatives to clinician-collected specimens for the diagnosis of human papillomavirus (HPV)^23^. A systematic review and meta-analysis conducted in 2015 on *Neisseria gonorrhoea* (NG) and *Chlamydia trachomatis* (CT) also reported that self-collected specimens were reliable for diagnostic testing^15^*.* Considering the potential impact of the self-sampling intervention on sexual and reproductive healthcare there is a need for an updated systematic review and meta-analysis sexual and reproductive healthcare. This is to foster improvements in clinical decision-making pertaining to sexual and reproductive healthcare provision. As such, the current study is an updated systematic review and meta-analysis on the accuracy of self-collected specimens compared to healthcare worker-collected specimens for STI diagnosis. This study will evaluate the diagnostic accuracy of self-sampling for STI diagnosis in studies conducted from 2015 onwards because a systematic review of a similar nature included studies up to 2015. The overarching aim of an updated systematic review is to ensure that the best evidence to inform clinical decision making and healthcare policy development for STI healthcare is provided.

## Methods

### Protocol and registration

The protocol for this study was submitted to the International Registration of Systematic Reviews (PROSPERO), with the registration number CRD42022341462. This study was guided by the Preferred Reporting Items for Systematic Review and Meta-analyses (PRISMA)^24^.

### Eligibility criteria

The Population, Intervention, Comparison, and Outcome (PICO) framework for determining the research question eligibility was followed. Studies were included if they: (a) assessed the accuracy of self-collected specimens against healthcare worker-collected specimens for STI diagnosis in women were included, (b) studies that used healthcare worker collected specimens as the reference or gold standard, (c) the study population comprised of specimens that had been tested for STIs including HPV, NG, CT, *Treponema pallidum* (syphilis), *Trichomonas vaginalis* (TV), and *Mycoplasma genitalium* (MG), (d) examined self-collected versus clinician-collected samples using different diagnostic assays including nucleic-acid-based assays, and manual methods that included wet mount, culture, and gram stain peer-reviewed studies published in 2015 and onwards to diagnose STIs. Data on investigations conducted on females was extracted from studies that include people of another gender. There were no language restrictions applied and studies with different study designs were included. Studies were excluded if: (a) the time of self-sampling and healthcare worker specimen collection exceeded three weeks due to the window period for seroconversion, (b) presented information on combined specimen results, (c) self-sampling was not conducted in females, (d) self-sampling and healthcare worker collected specimen was collected from different individuals.

### Index test

The diagnostic accuracy of self-collected specimens to diagnose STIs was evaluated against healthcare worker specimens. Self-collected specimens for STI diagnosis included vaginal swabs, urine, cervical swabs and tampons. The sensitivity and specificity of each diagnostic assay for each STI were evaluated.

## Reference standard

Healthcare worker-collected specimens for the diagnosis of STIs were used as the gold reference standard in this study.

### Search strategy

A systematic search of data was conducted in Cochrane, Medline, Scopus, Web of Science, and PubMed electronic databases (see Table 1). The search was limited to studies from 2015 onwards. The Principal Investigator (PI) developed the search strategy with an experienced librarian at the University of Pretoria. Medical Subject Headings (MeSH) terms were used to define our searches with Boolean operators (AND/OR) between search terms. The search terms used included but were not limited to (1) “Self-sampling” or “self-collected” or ‘self-administered” or “self-obtained” (2) “sexually transmitted infections” (3) “diagnostic specimens” or “diagnostic samples” (4) “women” or “females”. A hand search for grey literature was also conducted on the WHO website, the Department of Health South Africa (DoH SA), and the Open Grey website.

**Table 1:** Database search.

Date	Database	Keywords	Number of results retrieved
14 July 2021	Scopus	(TITLE-ABS-KEY ( sampling OR sample OR “self sampling” OR “self sample” OR “sti testing” OR “sti diagnosis” OR “sexually transmitted infections test*” OR “self-collect*” OR “sexually transmitted disease testing*” ) AND TITLE-ABS-KEY ( “Specimen Handling” ) AND TITLEABS-KEY (“Sexually Transmitted Disease*” OR “sexually transmitted infection*” ) AND TITLE-ABS-KEY ( wom*n OR female* OR girl* ) AND NOT TITLE-ABS-KEY ( aids OR “HIV Infections” OR hiv OR “human immunodeficiency virus” OR “acquired immunodeficiency syndrome” ) )	117
15 July 2021	Cochrane	(sampling OR sample OR “self sampling” OR “self sample” OR “sti testing” OR “sti diagnosis” OR “sexually transmitted infections test*” OR “selfcollect*” OR “sexually transmitted disease testing*”):ti,ab,kw (Word variations have been searched)	26
19 July 2021	PubMed	(((sampling[tw] OR sample[tw] OR “self sampling”[tw] OR “self sample”[tw] OR “sti testing”[tw] OR “sti diagnosis”[tw] OR “sexually transmitted infections test*”[tw] OR “self-collect*”[tw] OR “sexually transmitted disease testing*”[tw] AND (female[Filter])) AND (“Specimen Handling/methods”[Mesh] OR “Specimen Handling”[tw] AND (female[Filter]))) AND (“Sexually Transmitted Diseases, Bacterial”[Mesh] OR “Sexually Transmitted Diseases, Viral”[Mesh] OR “sexually transmitted infection*”[tw] OR “sexually transmitted disease*”[tw])) NOT (“HIV Infections”[Mesh] OR “HIV Infections”[tw])	213
19 July 2021	Wb of Science	((((ALL = (sampling OR sample OR “self sampling” OR “self sample” OR “sti testing” OR “sti diagnosis” OR “sexually transmitted infections test*” OR “self-collect*” OR “sexually transmitted disease testing*”)) AND ALL = ( “Sexually Transmitted Disease*” OR “sexually transmitted infection*” OR STI OR STD)) AND ALL = (wom*n OR female* OR girl*)) AND ALL = (“Specimen Handling” or “Specimen Collection” OR Specimen)) NOT ALL = (aids OR “HIV Infections” OR hiv OR “human immunodeficiency virus” OR “acquired immunodeficiency syndrome”)	311
21 July 2022	MEDLINE (EBSCO)	(((ALL = (sampl* OR “self sampl*” OR “sti test*” OR “sti diagnosis” OR “sexually transmitted infections test*” OR “self-collect*” OR “sexually transmitted disease test*”))) AND ALL = ( ) NOT ALL = (“)	140
26 Aug 2022	PubMed	(((sampling[tw] OR sample[tw] OR “self sampling”[tw] OR “self sample”[tw] OR “sti testing”[tw] OR “sti diagnosis”[tw] OR “sexually transmitted infections test*”[tw] OR “self-collect*”[tw] OR “sexually transmitted disease testing*”[tw]) AND (“Specimen Handling/methods”[Mesh] OR “Specimen Handling”[tw])) AND (“Sexually Transmitted Diseases, Bacterial”[Mesh] OR “Sexually Transmitted Diseases, Viral”[Mesh] OR “sexually transmitted infection*”[tw] OR “sexually transmitted disease*”[tw])) NOT (“HIV Infections”[Mesh] OR “HIV Infections”[tw]) Filters: Female, from 2021—2022	8
26 August 2022	Web of Science	((((ALL = (sampling OR sample OR “self sampling” OR “self sample” OR “sti testing” OR “sti diagnosis” OR “sexually transmitted infections test*” OR “self-collect*” OR “sexually transmitted disease testing*”)) AND ALL = ( “Sexually Transmitted Disease*” OR “sexually transmitted infection*” OR STI OR STD)) AND ALL = (wom*n OR female* OR girl*)) AND ALL = (“Specimen Handling” or “Specimen Collection” OR Specimen)) NOT ALL = (aids OR “HIV Infections” OR hiv OR “human immunodeficiency virus” OR “acquired immunodeficiency syndrome”)	28
26 August 2022	MEDLINE (EBSCO)	( sampling OR sample OR “self sampling” OR “self sample” OR “sti testing” OR “sti diagnosis” OR “sexually transmitted infections test*” OR “self-collect*” OR “sexually transmitted disease testing*” ) AND ( (MH “Sexually Transmitted Diseases + ”) OR “Sexually Transmitted Disease*” OR “sexually transmitted infection*” OR STI OR STD ) AND ( “Specimen Handling” OR (MH “Specimen Handling + ”) ) NOT ( (MH “HIV”) OR (MH “Acquired Immunodeficiency Syndrome”) OR aids OR “HIV Infections” OR hiv OR “human immunodeficiency virus” OR “acquired immunodeficiency syndrome” )	12
26 August 2022	Scopus	( TITLE-ABS-KEY ( sampling OR sample OR “self sampling” OR “self sample” OR “sti testing” OR “sti diagnosis” OR “sexually transmitted infections test*” OR “self-collect*” OR “sexually transmitted disease testing*” ) AND TITLE-ABS-KEY ( “Specimen Handling” ) AND TITLEABS-KEY ( “Sexually Transmitted Disease*” OR “sexually transmitted infection*” ) AND TITLE-ABS-KEY ( wom*n OR female* OR girl* ) AND NOT TITLE-ABS-KEY ( aids OR “HIV Infections” OR hiv OR “human immunodeficiency virus” OR “acquired immunodeficiency syndrome” ) )	7
26 August 2022	Cochrane	(sampling OR sample OR “self sampling” OR “self sample” OR “sti testing” OR “sti diagnosis” OR “sexually transmitted infections test*” OR “selfcollect*” OR “sexually transmitted disease testing*”):ti,ab,kw (Word variations have been searched)	0

### Study selection

Screening of studies suitable for inclusion in the systematic review and meta-analysis was conducted on the studies between 2015 and 2022. Since this systematic review stems from the findings of a scoping review which was conducted in 2021. Studies which had been screened for the scoping review from 2015 to 2021 were re-screened using eligibility criteria for the systematic review. To ensure the inclusion of studies conducted in 2022, the assisting librarian conducted a new search for studies that were published in 2022. An EndNote library was then created for all studies that were eligible for full-text screening. Thereafter, ZNJ and TD performed full-text screening of all studies that fulfilled the eligibility criteria of the systematic review and meta-analysis. NT resolved discrepancies that arose during full-text screening by ZNJ and TD. Thereafter, ZNJ and NT extracted data from studies found eligible for inclusion at the full-text screening stage. Thereafter, any disagreements were resolved by discussion until an agreement was reached. Study selection for the systematic review was guided by the PRISMA flowchart.

### Data extraction

ZNJ and NT independently extracted data from eligible studies using a data extraction tool that was designed to extract data from the included primary studies. The tool was piloted using 10% of the included studies and amended accordingly before final use. The extracted data was divided into two separate sections namely a section for basic qualitative information and another section for the quantitative outcomes of interest. Basic information extracted included author name(s) and year of publication, study title, study aims, study population, study design, sample size, eligibility criteria, reference standard specimen, type of self-collected specimen, type of laboratory assay, main findings, and conclusions. Data extracted for the section on the outcome of primary studies true positive, true negative, false positive, false negative, sensitivity and specificity, positive predictive value, negative predictive value, and evidence of agreement or concordance between self-collected and healthcare worker collected specimens. In some instances, the true negative, true positive false positive and false negative results were not available, and the relevant data was requested from the authors. A 2 × 2 table was produced based on the collected data. Any discrepancies that arose between the reviewers were discussed until a unanimous resolution was reached.

### Assessment of methodological quality

The Quality Assessment of Diagnostic Accuracy Studies 2 (QUADAS-2) tool for primary diagnostic accuracy studies, was utilised to assess the quality of all the included studies^25^. This tool consists of four main domains that include patient selection, index test, reference standard, and flow and timing^25^, which were adapted to the current study accordingly. To determine the risk of bias, signalling questions answered as “yes” “no” or “unclear”, were used in each phase^25^.

### Statistical analysis and data synthesis

For included studies in which sensitivity and specificity had been assessed and reported a meta-analysis of diagnostic accuracy was performed. The Review Manager (RevMan) software was used to conduct statistical analysis. The RevMan software was also used to calculate the pooled sensitivity, specificity, and diagnostic odds ratio with a 95% confidence interval. Cochran’s Q statistics were utilised to determine heterogeneity among the included primary studies. Statistical significance in all the analyses was calculated using the *p*-value where a *p*-value of < 0.05 indicated statistical significance.

### Ethical approval

Ethical clearance for the study was obtained from the University of Pretoria’s Faculty of Health Sciences Research Ethics Committee. The reference number is 136/2022. Participant consent was not applicable.

## Results

### Study selection and characteristics of included studies

**Figure 1:**
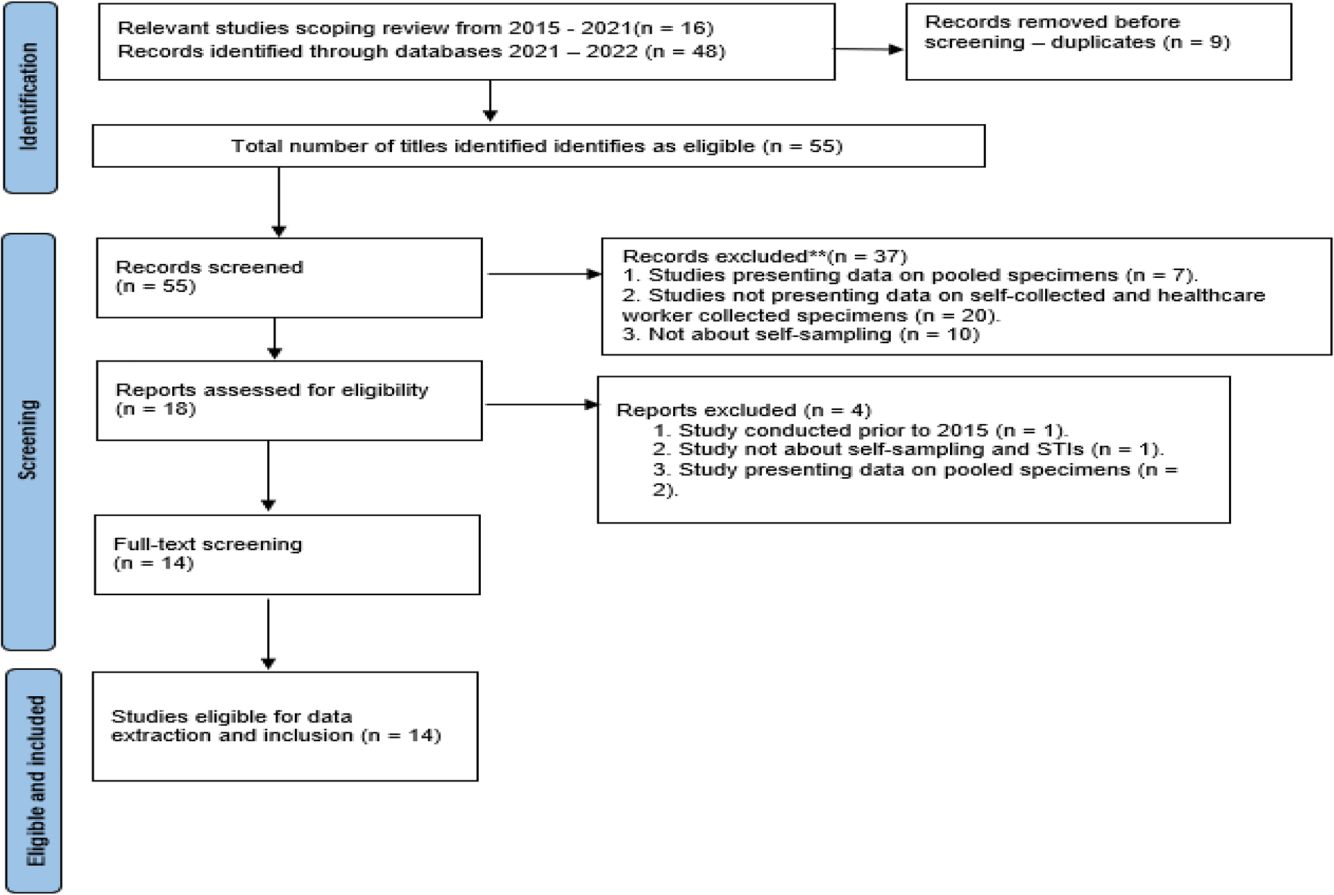
PRISMA flow diagram of the selection process of relevant studies.

Sixteen studies conducted in 2015, which were retrieved during a database search for the scoping review underwent title screening using the relevant eligibility criteria for the systematic review. For the new database search conducted by the librarian to ensure the inclusion of studies in Aug 2022, forty-eight search results were retrieved. Nine were duplicates, which left only thirty-nine eligible for title screening. The abstract screening was then conducted on fifty-five studies (thirty-nine plus sixteen studies). Post abstract screening, thirty-seven studies were excluded and only eighteen studies were eligible for data extraction. Reasons for exclusion were studies presenting data on pooled specimens, studies not presenting data on self-collected and healthcare worker collected specimens, and studies not about self-sampling STIs. Post full text screening of the studies only fourteen were eligible for inclusion in the systematic review. Four studies were excluded for being conducted before 2015, studies not about self-sampling, not about STIs, and a study presenting data on pooled specimens. Ultimately, data extraction was conducted on a total of fourteen studies (see Fig. 1 below). There was moderate agreement between the reviewers at full-text screening (*kappa* = *0.5*).

### Characteristics of included studies

The characteristics of included studies are all depicted in Table 2. Fourteen studies were included in the systematic review but not all of them were included in the meta-analysis. A large portion of the studies, five studies, were from the United States of America (USA)^26–30^, one study in Canada^31^, one in Haiti^32^, one in France^33^, one study in Saudi Arabia^34^, one in India^35^, one in the Republic of Korea^36^, one study in Kenya^37^, one in Chad^19^, one study in Ghana^38^. See Table 3 for quantitative characteristics of included studies. It is important to note that some of the sensitivity and specificity measurements were obtained from the articles as calculated by the authors. However, where the measurements were not available, the researchers calculated using data that was already available on the manuscripts and original data obtained from authors of some of the included studies. Furthermore, for studies where this information was not available at all, it was not reported.

**Table 2:** Characteristics of included studies.

Disease	Author, year published	Country of study	Study design	Study population (sex) and samples size	Mode of instruction for self-collection	Location of self-collection	Specimen and testing		
							Specimen (healthcare worker collected/self-collected	Diagnostic platform Automated (run on instrument)/manual (manual method used)	Assay type
CT	Arias et al. 2016 ^31^	Canada	Cross sectional	Female -189	Demonstration of collection method and self-collection had collection instructions	Study clinic	Vaginal swab/vaginal swab	Automated	NAAT Aptima Combo 2
	Camus et al. 2021 ^33^	France	Cross sectional	Female = 1028	Instructions provided	Study clinic	Vaginal/cervical classical sampling/vaginal swab	Automated	COBAS—Roche Diagnostics Kits
	De Marais et al. 2018 ^27^	USA	Clinical trial	Female = 193	Participants were instructed by a study nurse	Home and clinic	Cervical swab/cervicovaginal swab	Automated	Aptima Combo2
	Lockhart et al. 2018 ^37^	Kenya	Cross-sectional	Female = 350	Participants were instructed verbally	Study clinic	Cervical swab/cervicovaginal swab	Automated	Aptima combo assay
	Nodjikouambaye et al. 2019 ^19^	Chad	Cross-sectional	Female = 271	Training on specimen collection	Study clinic	Endocervical swab/veil sample	Automated	Multiplex real-time PCR—Allplex STI Essential Assay
	Van Der Pol et al. 2019 ^30^	USA	Cross sectional	Female = 3860	Not indicated	Not indicated	Vaginal swab/vaginal swab, urine	Automated	NAAT—COBAS NG/CT test—the BD ProbeTec CT Qx and GC Qx amplified DNA assay; Aptima Combo 2 CT/NG; and the Abbott m2000 RealTime CT/NG assay
HPV	Boggan et al. 2015 ^32^	Haiti	Cohort	Female = 1836	Orientation by a study nurse	Study clinic	Cervical swab/vaginal swab	Automated	Hybrid Capture 2 High-Risk HPV DNA Test
	De Marais et al. 2018 ^27^	USA	Clinical trial	Female = 193	Participants were instructed by a study nurse	Home and clinic	Cervical swab/cervicovaginal swab	Automated	Aptima HPV assay
	Kim et al. 2020 ^36^	Korea	Cross sectional	Female = 151	Digital and written instructions provided	Study clinic	Cervical swab/vaginal swab	Automated	multiplex real-time PCR Anyplex II HPV28 Detection assay
	McLarty et al. 2019 ^28^	USA	Cross sectional	Female = 174	Individual instructions were provided	Home and study clinic	Cervical swab/tampon, vaginal swab	Automated	Roche Cobas HPV method
	Obiri-Yeboah et al., 2017 ^38^	Ghana	Cross sectional	Female = 333	Instructed on how to obtain a specimen	Study clinic	Cervical swab/vaginal swab	Automated	careHPV assay
MG	Camus et al. 2021 ^33^	France	Cross sectional	Female = 1028	Instructions provided	Study clinic	Vaginal/cervical classical sampling/vaginal swab	Automated	TIB MOLBIOL LightMix—PCR Roche Diagnostics
	De Marais et al. 2018 ^27^	USA	Clinical trial	Female = 193	Participants were instructed by a study nurse	Home and clinic	Cervical swab/cervicovaginal swab	Automated	Aptima analyte-specific reagent-based assay
	Lockhart et al. 2018 ^37^	Kenya	Cross-sectional	Female = 350	Participants were instructed verbally	Study clinic	Cervical swab/cervicovaginal swab	Automated	Aptima combo assay
	Nodjikouambaye et al. 2019 ^19^	Chad	Cross-sectional	Female = 271	Training on specimen collection	Study clinic	Endocervical swab/veil sample	Automated	Multiplex real-time PCR—Allplex STI Essential Assay
*NG*	Arias et al. 2016 ^31^	Canada	Cross sectional	Female = 189	Demonstration of collection method and self-collection had collection instructions	Study clinic	Vaginal swab/vaginal swab	Automated	NAAT Aptima Combo 2
	Barbee et al. 2021 ^26^	USA	Cross-sectional	Female = 89	Not indicated	Study clinic	Endocervical swab/vaginal swab	Manual and automated	Culture and NAAT Aptima Combo 2
	Camus et al. 2021 ^33^	France	Cross sectional	Female = 1028	Instructions provided	Study clinic	Vaginal/cervical classical sampling/vaginal swab	Automated	COBAS—Roche Diagnostics Kits
	De Marais et al. 2018 ^27^	USA	Clinical trial	Female = 193	Participants were instructed by a study nurse	Home and clinic	Cervical swab/cervicovaginal swab	Automated	Aptima Combo2
	Lockhart et al. 2018 ^37^	Kenya	Cross-sectional	Female = 350	Participants were instructed verbally	Study clinic	Cervical swab/cervicovaginal swab	Automated	Aptima combo assay
	Nodjikouambaye et al. 2019 ^19^	Chad	Cross-sectional	Female = 271	Training on specimen collection	Study clinic	Endocervical swab/veil sample	Automated	Multiplex real-time PCR—Allplex STI Essential Assay
	Van Der Pol et al. 2019 ^30^	USA	Cross sectional	Female = 3860	Not indicated	Not indicated	Vaginal swab/vaginal swab, urine	Automated	NAAT—COBAS NG/CT test—the BD ProbeTec CT Qx and GC Qx amplified DNA assay; Aptima Combo 2 CT/NG; and the Abbott m2000 Real-Time CT/NG assay
TV	Camus et al. 2021 ^33^	France	Cross sectional	Female = 1028	Instructions provided	Study clinic	Vaginal and cervical swabs/vaginal swab	Automated	TIB MOLBIOL LightMix—PCR Roche Diagnostics
	De Marais et al. 2018 ^27^	USA	Clinical trial	Female = 193	Participants were instructed by a study nurse	Home and clinic	Cervical swab/cervicovaginal swab	Automated	Aptima TV assay
	Hawash et al. 2021 ^34^	Saudi Arabia	Cross sectional	Female = 174	Instructions were provided and sample collection was done in the presence of medical personnel	Study clinic	Vaginal swab/vaginal swab	Manual, and automated	OSOM TV rapid test, wet prep, TV DNA PCR
	Khan et al. 2019 ^35^	India	Cross-sectional	Female = 550	Participants were given instructions	Study clinic	Vaginal swab/vaginal swab	Manual	Trichomonas culture
	Lockhart et al. 2018 ^37^	Kenya	Cross-sectional	Female = 350	Participants were instructed verbally	Study clinic	Cervical swab/cervicovaginal swab	Automated	Aptima combo assay
	Nodjikouambaye et al. 2019 ^19^	Chad	Cross-sectional	Female = 271	Training on specimen collection	Study clinic	Endocervical swab/veil sample	Automated	Multiplex real-time PCR—Allplex STI Essential Assay
	Schwebke et al., 2018 ^29^	USA	Cross sectional	Female = 1867	Not indicated	Study clinic	Cervical swab/vaginal swab	Manual, and automated	In Pouch TV broth culture and Aptima NAAT for TV

CT = *Chlamydia trachomatis;* NG = *Neisseria gonorrhoea;* TV = Trichomonas vaginalis; HPV = Human papillomavirus; DNA = Deoxyribonucleic acid; PCR = Polymerase Chain Reaction; Veil sample = self-collection device for cervicovaginal fluid collection.

**Table 3:** Quantitative characteristics of included studies.

Disease	Author, year published	TP	FP	TN	FN	PPV (%)	NPV (%)	Cohen’s kappa/concordance (%)	Healthcare worker Vs self-collected		Self-collected only		Healthcare worker collected only	
									Sensitivity (%)	Specificity (%)	Sensitivity (%)	Specificity (%)	Sensitivity (%)	Specificity (%)
*Chlamydia trachomatis*	Arias et al. 2016 ^31^	5	10	159	6	33	96	98.4	50	94.4	–	–	–	–
	Camus et al. 2021 ^33^	33	1	994	0	97.06	100	99.9%	100 (NA)	99.9 (99.7–100)	–	–	–	–
	Nodjikouambaye et al. 2019 ^19^	126	12	90	4	91	95	92.8%	97 (80.7–93.3)	88 (80.7–93.3)	–	–	–	–
	Van Der Pol et al. 2019 ^30^	119	17	1769	1	87.5	99.9	–	–	–	99.2 (95.4–99.4)	99 (99.4–99.9)	98.6 (95.2–99.6)	99.1 (98.6–99.4)
Human papilloma virus	De Marais et al. 2018 ^27^ (HSIL)	–	–	–	–	–	–	0.66	–	–	100 (NA)	88.9 (83.6–93.0)	100 (NA)	90 (84.8–93.9)
	De Marais et al. 2018 ^27^ (CIN 2)	–	–	–	–	–	–	0.66	–	–	100 (NA)	91.1% (86–94.8)	100 (NA)	92.2% (87.3–95.7)
	Obiri-Yeboah et al., 2017 ^38^	–	–	–	–	–	–	94.2	92.6 (85.3–97.0)	95.9 (89.8–98.8)	–	–	–	–
*Mycoplasma genitalium*	Camus et al. 2021 ^33^	14	0	0	1014	100	100	100%	100 (NA)	100 (NA)	–	–	–	–
	Nodjikouambaye et al. 2019 ^19^	126	12	90	4	91	95	–	97 (80.7–93.3)	88 (80.7–93.3)	–	–	–	–
*Neisseria gonorrhoea*	Arias et al. 2016 ^31^	0.8	4	180	1	17	99	98.4%	40	98.4	–	–	–	–
	Camus et al. 2021 ^33^	7	1020	1021	1	0.68	99.9	99.9%	85.7 (59.9–100)	100 (NA)	–	–	–	–
	Nodjikouambaye et al. 2019 ^19^	126	12	90	4	91	95	86%	97 (80.7–93.3)	88 (80.7–93.3)	–	–	–	
	Van Der Pol et al. 2019 ^30^	28	0	1903	5	84.5	100	–	–	–	100 (87.9–99.9)	99.7 (99.3–99.9)	100 (87.9–100)	99.7 (99,4–99.9)
Trichomoniasis vaginalis	Camus et al. 2021 ^33^	9	1015	1015	0	0.88	100	99.8%	100 (NA)	99.8 (99.5–100)	–	–	–	–
	Hawash et al. 2021 ^34^	15	2	127	5	88.2	100	97.9%	–	–	83.3 (58.5–96.4)	98.4 (94.5–99.8)	88.8 (65.2–98.6)	100 (97.1–100)
	Khan et al. 2019 ^35^	3	0	547	0	100	100	100%	100	100	–	–	–	–
	Nodjikouambaye et al. 2019 ^19^	126	12	90	4	91	95	92.8	97 (80.7–93.3)	88 (80.7–93.3)	–	–	–	–
	Schwebke et al., 2018 ^29^ (InPouch)	156	37	1593	5	80.8	99.7	–	96.90 (92.9–99.9)	97.70 (96.9–98.4)	–	–	–	–
	Schwebke et al., 2018 ^29^ (Aptima assay)	186	5	1593	7	96.4	99.7	–	97.4 (94.0–99.1)	99.6 (99.1–99.8)	–	–	–	–
	Schwebke et al., 2018 ^29^ (PIS)	186	7	1591	7	96.4	99.6	–	96.4 (92.7–98.5)	99.6 (99.1–99.8)	–	–	–	–
	Schwebke et al., 2018 ^29^ (Xpert vs PIS)	186	7	1591	7	96.4	99.6	–	96.4 (92.7–98.5)	99.6 (99.1–99.8)	–	–	–	–

TP = True positive; FN = False positive; TN = True negative; FN = False negative; PPV = Positive predictive value; NPV = Negative predictive value; PIS = patient infected status.

The characteristics of the included studies were further divided into sub-groups for meta-analysis for each STI as outlined in the following sections:

#### Chlamydia

**Figure 2:**
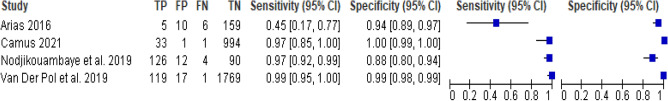
Forest plot of chlamydia studies that compared self-collected vaginal swabs with healthcare worker collected cervical and vaginal specimens.

**Figure 3:**
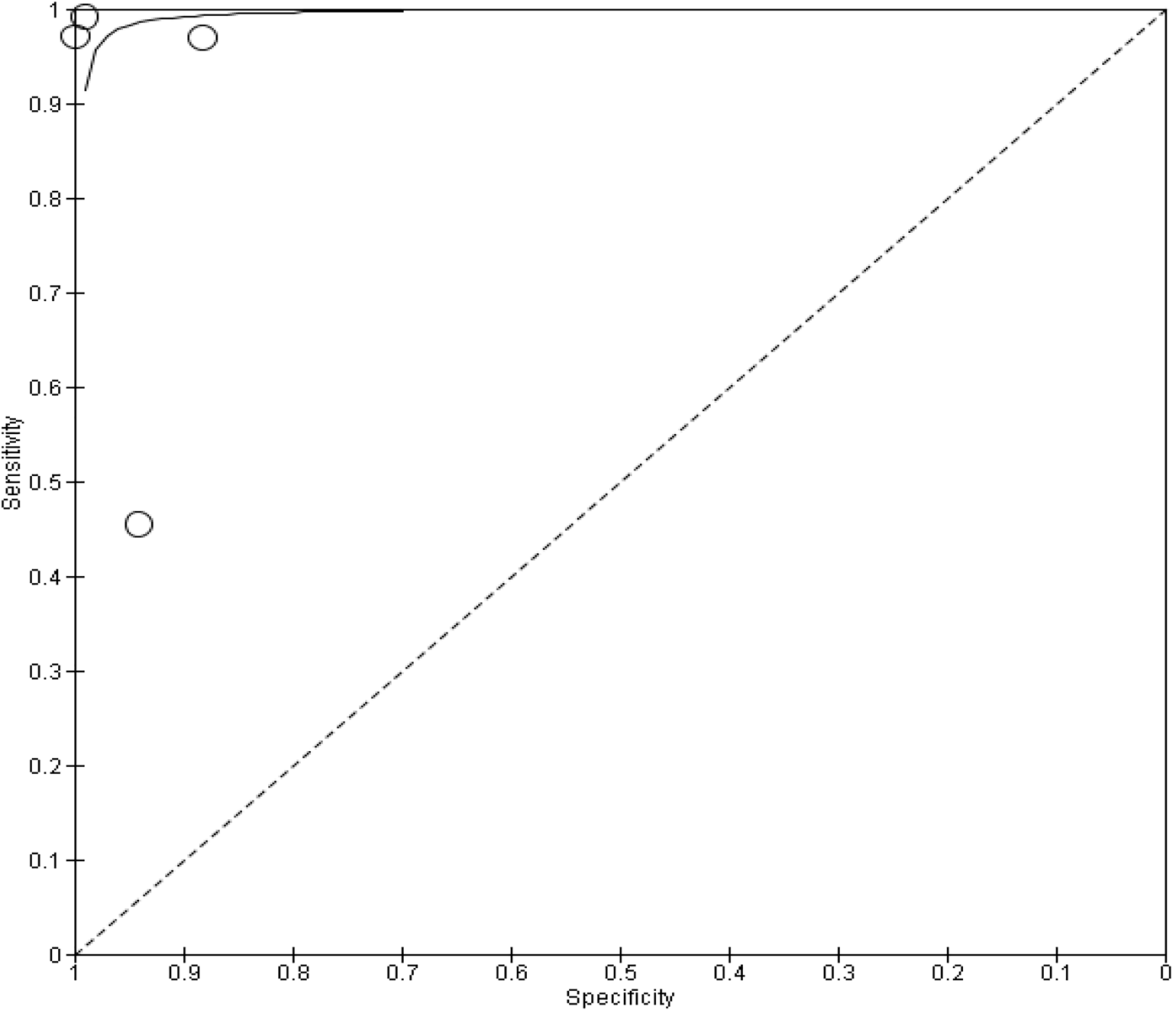
SROC depicting diagnostic accuracy of included studies for chlamydia.

A total of six studies compared the diagnostic accuracy of self-collected specimens to healthcare worker collected specimens in females^19,27,30,31,33,37^. Five of the studies were conducted in a clinic^19,27,31,33,37^, and study location was not reported for one of the studies^30^. Of these six studies, three of them compared healthcare worker collected vaginal swabs to self-collected vaginal swabs^30,31,33^. In two of the studies healthcare workers collected cervical swabs were compared to self-collected cervicovaginal swabs^27,37^. Only one study compared healthcare worker collected endocervical swabs to self-collected veil specimens^19^. STI testing was performed using automated NAAT based assays. All six studies were cross-sectional studies. In five of the studies, research participants had received instructions on how to self-collect specimens for testing^19,27,31,33,37^, and in one study the research participants did not receive any instructions^30^. The number of research participants in the studies ranged from 189 to 3860. Only four of the six studies were included in the subgroup meta-analysis^19,30,31,33^. Out of the two excluded studies, one study was excluded because only agreement data was reported and the other parameters were not reported^37^. Similarly, the other study only reported sensitivity and specificity data^27^. Figure 2 presents research findings for the subgroup analysis of four studies, where the summary estimate for sensitivity was 0.85 (95% Confidence Interval 0.77–0.92), while specificity was 0.95 (95% Confidence Interval 0.91–0.98). The SROC plot (Fig. 3) is a depiction of the pooled sensitivity and specificity of the studies.

The studies show statistical significance in the studies, but there is moderate evidence of heterogeneity among the studies. The diagnostic tests have a good discriminatory ability to differentiate between individuals with and without chlamydia (Table 4).

**Table 4:** Heterogeneity and statistical significance for CT.

Item	Result
*P* value	0.02. The result is significant at *p* < 0.05
Cochran’s Q (heterogeneity)	9.82
DOR	7.78

#### Human papilloma virus

Five studies compared the diagnostic accuracy of healthcare worker collected specimens with self-collected specimens to diagnose HPV^27,28,32,36,38^. Three of the studies compared healthcare worker collected cervical swabs were compared to self-collected vaginal swabs^32,36,38^, while one study compared healthcare worker collected cervical swabs with self-collected tampons and vaginal swabs^28^, and another study compared healthcare worker collected cervical swab with self-collected cervicovaginal swabs^27^. All the studies were conducted in a research clinic. The sample size of the studies ranged from 151 to 1836 study participants. Study participants received instructions on how to self-collect their specimens for STI diagnosis, prior to specimen collection. NAAT based diagnostic assays were used in all the studies. Four of the studies were cross-sectional studies^28,32,36^, and only one was a clinical trial^27^. In one study, the sensitivity and specificity of self-collected specimens was 100 and 88.9% respectively, while healthcare worker collected diagnostic result sensitivity and specificity were 100 and 90% respectively^27^. In another study, the sensitivity and specificity of self-collected specimens compared to healthcare worker collected specimens were 92.6 and 95.9% respectively^38^. One study reported the sensitivity of self-collected specimens as 100%^36^. Another study reported the sensitivity and specificity of only self-collected swab as 86 and 94% respectively, while for the self-collected tampon it was 77 and 100% respectively^28^. Another study reported sensitivity results of self-collected specimens as 89.1% and sensitivity of healthcare workers collected specimens as 87.9%^32^. However, a sub-group meta-analysis was not performed because the relevant data for TN, FN, TP and FP was not available.

#### Mycoplasma genitalium

**Figure 4:**
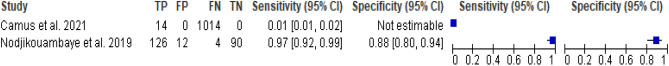
Forest plot of MG studies that compared self-collected vaginal swabs with healthcare worker collected cervical and vaginal specimens.

Out of the four studies that investigated MG infection, two studies compared self-collected cervicovaginal swabs with healthcare worker collected cervical swabs^27,37^; one study compared healthcare worker collected vaginal and cervical swabs with self-collected vaginal swabs^33^, and another one compared healthcare worker collected endocervical swabs with self-collected veil specimens^19^. Diagnostic testing was performed using NAAT based assays in all the studies. All the studies were conducted in clinics. In all the studies, research participants received instructions on how to self-collect specimens before collecting their own specimens. The sample size ranged from 193 to 1028 participants. All studies were cross-sectional. Only two of the included studies had sufficient data for a meta-analysis for this subgroup^19,33^. Figure 4 presents the analysis of the two studies where the summary estimate for sensitivity was 0.49 (95% Confidence Interval 0.39–0.58) and for specificity was 0.88 (95% Confidence Interval 0.81–0.94).

**Figure 5:**
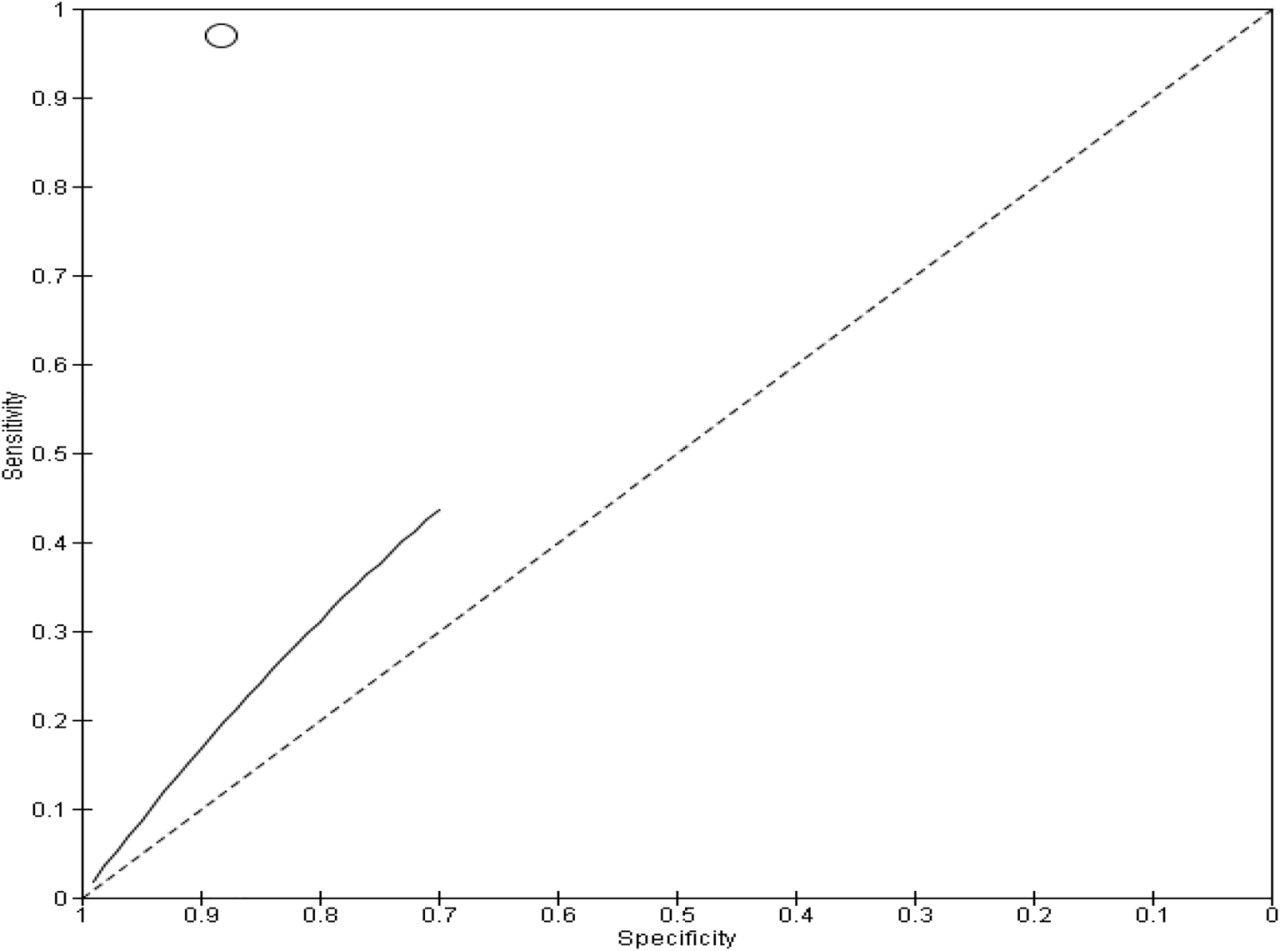
SROC depicting diagnostic accuracy of MG in included studies.

Presented below in Fig. 5 is the SROC plot depicting the diagnostic accuracy of the studies in this subgroup.

The sub-group meta-analysis suggests that the accuracy of the diagnostic test may vary across studies, with poor sensitivity in one study and poor specificity in the other. However, overall, the test shows a moderate to high diagnostic accuracy, as indicated by the high DOR value (Table 5).

**Table 5:** Heterogeneity and statistical significance for MG.

Item	Result
*P* value	0.001. The result is significant at *p* < 0.05
Cochran’s Q (heterogeneity)	15.50
DOR	21.7

#### Gonorrhoea

**Figure 6:**
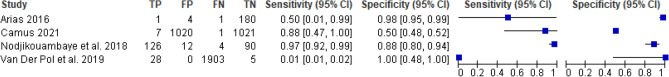
Forest plot of gonorrhoea studies that compared self-collected vaginal swabs with healthcare worker collected cervical and vaginal specimens.

Seven studies investigated the diagnostic accuracy of self-collected specimens in comparison to healthcare worker collected specimens in diagnosing NG. Six of these studies were cross-sectional^19,26,30,31,33,37^, and only one was a clinical trial^27^. The sample size of the studies ranged from 89 to 3860. Laboratory diagnosis was performed using automated NAAT based assays in all the studies, and one of the studies also used manual diagnostic methods^26^. Six studies reported that specimen collection had occurred at research clinics^19,26,27,31,33,37^, and one study did not indicate^30^. In six of the studies, research participants received instructions before specimen collection^19,26,27,31,33,37^, but in one study there was no report about whether research participants had been instructed how to self-collect their specimen^30^. Two studies compared diagnostic accuracy in healthcare worker collected vaginal swabs to self-collected vaginal swabs^30,31^. One study compared self-collected vaginal swabs to cervical and vaginal swabs collected by healthcare workers^33^. Two studies compared diagnostic accuracy in self-collected cervicovaginal swabs and healthcare worker collected cervical swabs^27,37^. In one study diagnostic accuracy is compared between healthcare worker collected endocervical swabs with self-collected vaginal swabs^26^. Another study compared diagnostic accuracy in self-collected veil specimens with healthcare worker collected endocervical swabs^19^. Figure 6 below presents summary estimates for the sensitivity and specificity of diagnostic accuracy of healthcare worker collected specimens compared to self-collected specimens. The summary estimate for sensitivity and specificity is 0.59 (95% Confidence Interval 0.49–0.68) and 0.84 (95% Confidence Interval 0.76–0.91).

**Figure 7:**
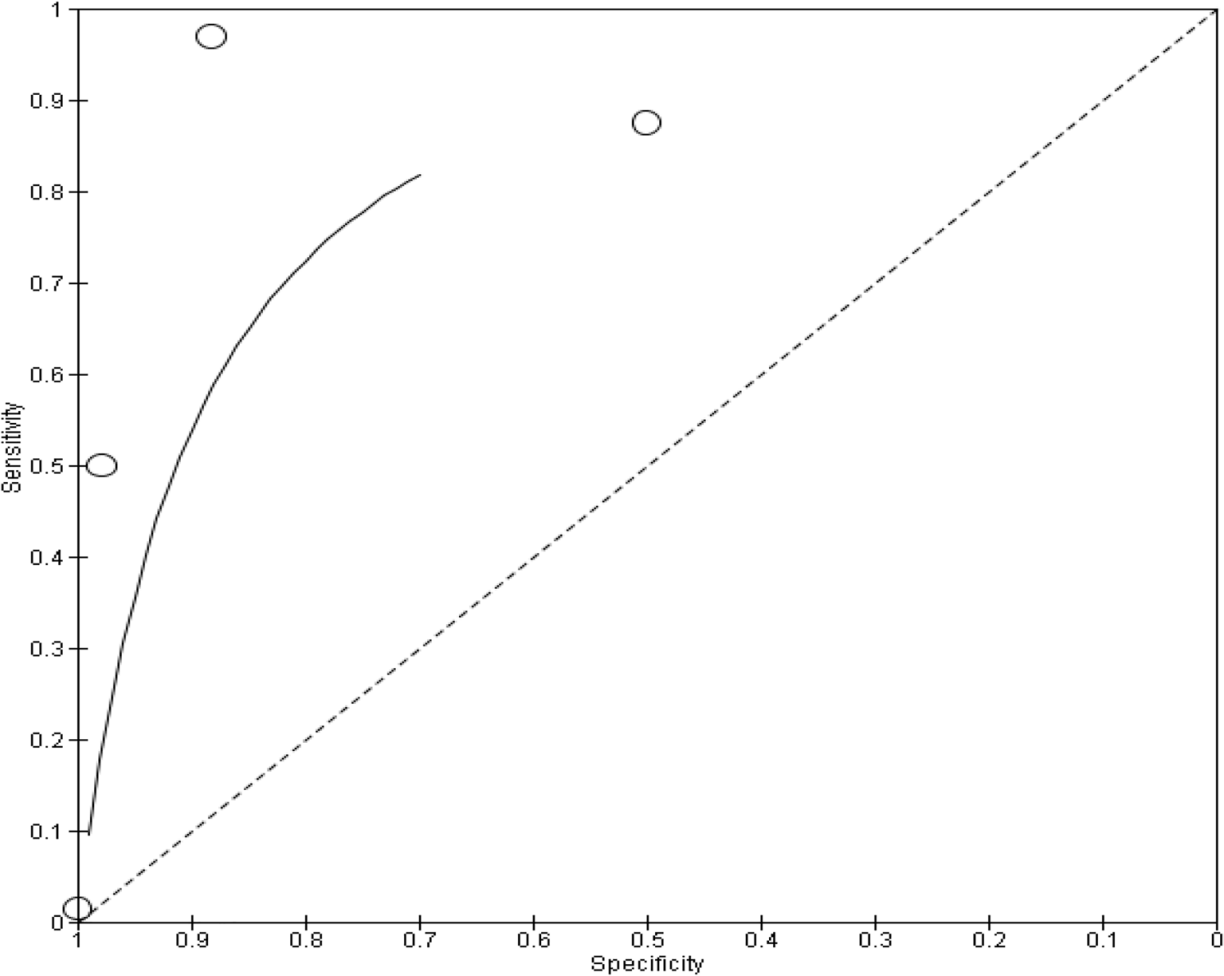
SROC depicting diagnostic accuracy of NG in included studies.

Presented below in Fig. 7 is the SROC plot depicting the diagnostic accuracy of the studies in this subgroup.

The Cochran's Q test shows significant heterogeneity among the studies at 17.156. The diagnostic odds ratio of 2.579 suggests that the overall accuracy of the diagnostic test is low to moderate. The *p*-value indicates statistical significance (Table 6).

**Table 6:** Heterogeneity and statistical significance for NG.

Item	Result
*P* value	0.0006. The result is significant at *p* < 0.05
Cochran’s Q (heterogeneity)	17.156
DOR	2.579

#### Trichomoniasis

**Figure 8:**
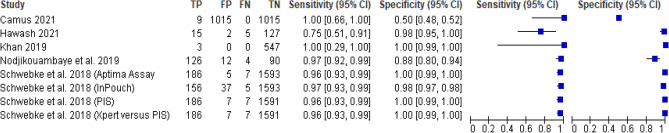
Forest plot of TV studies that compared self-collected vaginal swabs with healthcare worker collected cervical and vaginal specimens.

Seven studies investigated the diagnostic accuracy of self-collected specimens in comparison to healthcare worker collected specimens in diagnosing trichomoniasis. Six of the studies were cross-sectional^19,29,33–35,37^, and one study was a clinical trial^27^. Four studies utilised automated NAAT-based assays^19,27,33,37^, one study used manual testing methods^35^, while two studies used both automated NAAT assays and manual methods for TV diagnosis^29,34^. Study participants in all the studies collected their specimens at the research clinics. In five of the studies the participants received instructions on how to self-collect specimens before collecting their specimens^19,27,33–35,37^, and in one study this was not reported^29^. The sample size of research participants ranged from 174 to 1867. One study compared the diagnostic accuracy of healthcare worker collected vaginal and cervical swabs with self-collected swabs^33^. Two studies compared healthcare worker collected cervical swabs with self-collected vaginal swabs^29,37^. One study compared endocervical swabs collected by healthcare workers with self-collected veil specimens^19^. Two studies compared diagnostic accuracy between self-collected vaginal swabs with healthcare worker collected vaginal swabs^34,35^. Only one study compared healthcare worker collected endocervical swabs with self-collected vaginal swabs^19^. Figure 8 below presents summary estimates for the sensitivity and specificity of diagnostic accuracy healthcare worker collected specimens compared to self-collected specimens.

**Figure 9:**
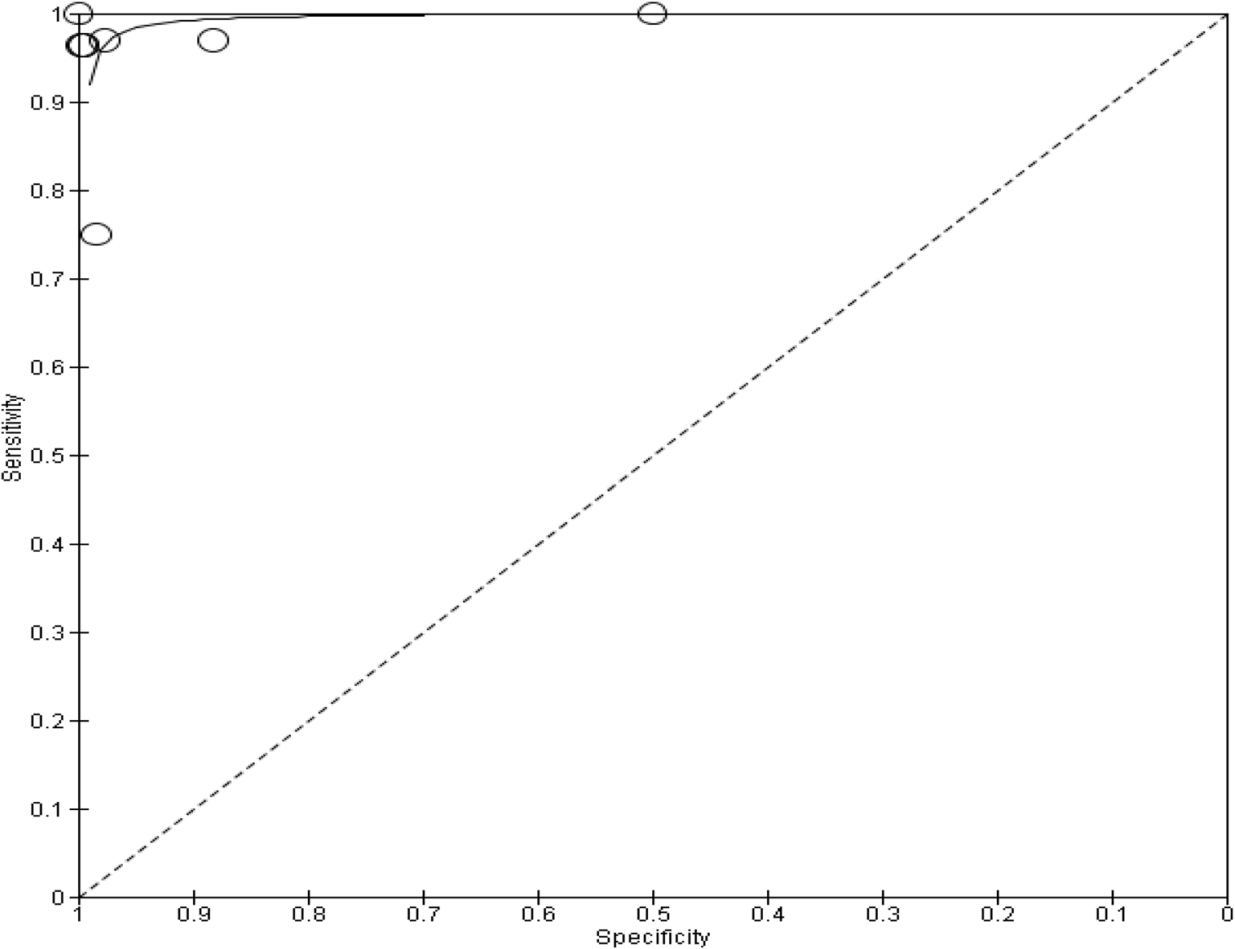
SROC depicting diagnostic accuracy of TV in included studies.

The summary estimate for sensitivity and specificity is 0.94 (95% Confidence Interval 0.89–0.98) and 0.91 (95% Confidence Interval 0.85–0.96) respectively and it is depicted on the SROC in Fig. 9 below. Additionally, Fig. 9 depicts the diagnostic accuracy of the studies in this subgroup.

The Cochran's Q test result shows that there is significant heterogeneity among the studies and the diagnostic test is moderately accurate in identifying patients with disease (Table 7).

**Table 7:** Heterogeneity and statistical significance for TV.

Item	Result
*P* value	0.001. The result is significant at *p* < 0.05
Cochran’s Q (heterogeneity)	25.15
DOR	20.02

### Methodological quality of studies

Table 8 below depicts the risk of bias and applicability assessment of included studies using the QUADAS-2 tool used to assess quality^25^. The domains of the QUADAS-2 tool are patient selection, index test, reference standard, and flow and timing. Patient selection outlines the process of selecting study participants in the primary studies which includes setting, presentation, prior testing, and intended use of index test; index test describes how the test of interest was conducted and interpreted; reference standard describes how the standard test was conducted and interpreted, and flow and timing describe excluded studies and intervals between the index and reference tests^25^. For the current study, the index test is designated as the self-collected specimens, while the reference test refers to the healthcare worker collected specimens.

**Table 8:**
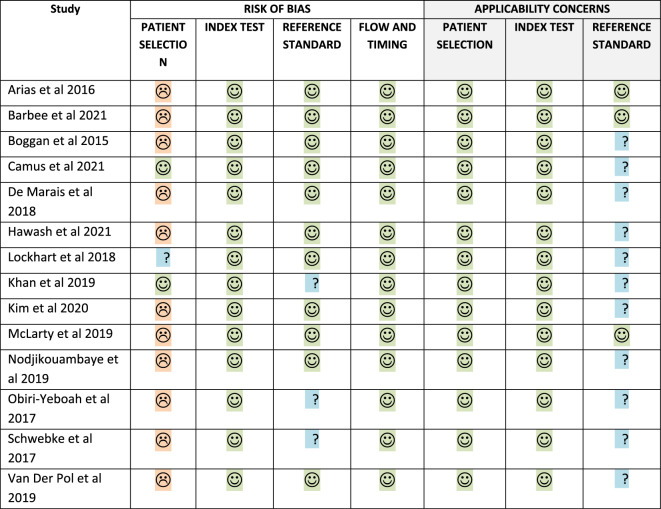
QUADAS-2 summary of methodological assessment.

**Figure 10:**
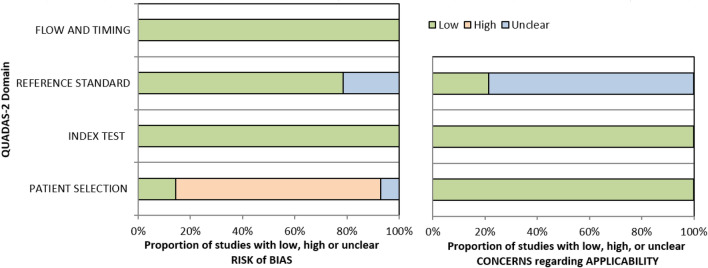
Assessment of included studies using QUADAS-2.

For the majority of the studies, the sampling approach utilised was convenience sampling and not random or consecutive sampling which are the options available in the patient selection domain. Although convenience sampling was used for most of the studies and therefore introduced a high-risk bias, that is unlikely to interfere with the diagnostic accuracy of self-sampling and healthcare worker collected specimens. The reference standard domain and flow and timing domains were found to mostly be at low risk of bias in all the studies. Concerning applicability, all studies were at low risk of bias. However, regarding the applicability of the reference standard, it was unclear for most studies. The graphical results of the included studies from the QUADAS-2 quality assessment tool are indicated in Fig. 10.

## Discussion

This study compared the diagnostic accuracy of self-collected specimens to healthcare worker collected specimens for diagnosing CT, HPV, MG, NG, syphilis, and TV in females. No studies on syphilis fulfilled the eligibility criteria for inclusion in this review. For CT, six studies were included in the analysis, out of which four were included in the subgroup meta-analysis. The summary estimate for sensitivity was 0.85 (0.77–0.92), while specificity was 0.95 (0.91–0.98). For HPV, five studies were included, and there was insufficient data to perform a sub-group meta-analysis. However, the sensitivity and specificity of self-collected specimens of the individual studies compared to healthcare worker collected specimens varied between studies, with sensitivity ranging from 86 to 100%, and specificity ranging from 88.9% to 100%. For MG, four studies investigated diagnostic accuracy, and two studies had sufficient data for a sub-group meta-analysis. The summary estimate for sensitivity was low at 0.49 (0.39–0.58), while specificity was 0.88 (0.81–0.94). For NG, seven studies were included in the analysis, and four studies were included in the sub-group meta-analysis. The pooled sensitivity and specificity estimate was 0.59 (0.49–0.68) and 0.84 (0.76–0.91) respectively.

In the case of CT and NG, it is important to note that the low sensitivity and high specificity are comparable to previous findings^15^. For TV, seven studies investigated diagnostic accuracy, and four studies were included in the sub-group meta-analysis. The results of the meta-analysis showed that self-collected specimens have high sensitivity and specificity for the diagnosis of trichomoniasis, with a summary estimate for sensitivity and specificity of 0.94 (0.89–0.98) and 0.91 (0.85–0.96), respectively.

The study found that there was significant heterogeneity among the studies. This may be attributed to differences in the methods used to collect and test specimens across the different studies. The DOR results indicated that the diagnostic tests used in the studies had a good ability to differentiate between individuals with and without CT, HPV, NG, MG and TV. The study also presented a SROC curve to visualize the sensitivity and specificity of all included studies, with most points falling between 0.9 and 1.00 on the y-axis (sensitivity), indicating better performance in distinguishing between the presence and absence of infection.

The QUADAS-2 tool was used to assess the quality of the included studies, and it showed that a majority of them used convenience sampling to select patients. Although this sampling method can increase the risk of bias, it did not appear to affect the diagnostic accuracy of self-collected specimens and specimens collected by healthcare workers. Most of the included studies had a low risk of bias in the index test, reference standard, flow, and timing domains. Overall, the included studies introduced minimal bias, which enhances the quality of the research findings. Study screening, selection, and data extraction were conducted systematically to ensure the most suitable studies were included in the review. A comprehensive approach to reviewing existing evidence on the diagnostic accuracy of self-collected specimens versus those collected by healthcare workers was employed. Only peer-reviewed and published studies were included to ensure reliable results. Some of the included studies utilized convenience sampling, which may have introduced bias in the patient selection process.

Since we classified healthcare worker collected specimens as the gold-standard diagnostic accuracy was presumed to be 100%. For CT the healthcare worker collected sensitivity ranged between 50 and 100%, while specificity was 88 and 99.2%; for MG sensitivity ranged between 97 and 100%, while specificity was 88 and 100%; NG sensitivity ranged between 40 and 97%, while specificity was 88 and 100%; and TV sensitivity ranged between 96 and 100%, while specificity was 88 and 100%.

The results indicate that self-collected specimens are a comparative alternative to healthcare worker collected specimens for STI testing. This is in keeping with previous studies that advocate for the use of self-sampling interventions as alternative tools to enable and promote screening of STIs even in asymptomatic patients and resource- limited settings^15,39^. These findings have important implications for STI testing, particularly in settings where access to healthcare workers may be limited or where stigma and embarrassment may prevent individuals from seeking testing.

### Limitations

The lack of eligible studies for syphilis and insufficient study data for meta-analysis in HPV limits the comprehensiveness of the review. There was significant heterogeneity among included studies, likely due to varying specimen collection and testing methods, which introduced variability and challenges with generalizability of the findings. Despite efforts to minimise bias during data analysis, the use of convenience sampling in most studies introduced potential bias in patient selection. Assuming the accuracy of the gold standard of healthcare worker-collected specimens may not fully capture variability in sensitivity and specificity among these samples. Conversely, the wide range of sensitivity and specificity values across individual studies underscores the complexity of interpreting overall diagnostic accuracy. Lastly, it is important to consider that the findings of this study may not be generalizable to resource-limited settings where access to healthcare workers and testing facilities differs.

## Conclusion

This study presents evidence of the accuracy of self-collected specimens when used to diagnose STIs in females. The meta-analysis findings highlight that the diagnostic accuracy of self-collected specimen to diagnose STIs in females is comparable with that of healthcare worker collected specimens. When considering the global burden of STIs on the public health system, such findings are an indication of how self-sampling for STI diagnosis could be used to improve STI management services across the globe. Although much evidence exists on the use of this intervention in high-income countries^22^, the researchers hope that the findings of this study will capture the attention of governments in LIMCs and cause them to see their need for it. Furthermore, the potential of self-sampling interventions to improve screening of asymptomatic STIs must be recognized and utilized as a tool to fulfil goal 3 of the sustainable development goals which is targeted at treating and improving access to quality healthcare for all people across the globe. The study is limited in that the investigation of diagnostic accuracy of self-collected specimens was only conducted on females. Therefore, the findings are not representative of self-collected specimens among a broader and more diverse population. We, therefore, recommend a future study to investigate the accuracy of self-collected specimens for diagnosing a wide range of STIs in a more diverse and broader population.

### Supplementary Information

Supplementary Information.

## Data Availability

All data generated or analysed during this study are included in this manuscript [and its supplementary information files].

## Abbreviations

CT: *Chlamydia trachomatis*
CIN2: Cervical intraepithelial neoplasia 2
DOR: Diagnostics Odds Ratio
DoH SA: Department of Health South Africa
FP: False positive
FN: False negative
HPV: Human papilloma virus
HSIL: High grade squamous intraepithelial lesion
LMICs: Low-and-middle-income countries
MeSH: Medical subject headings
MG: *Mycoplasma genitalium*
NAAT: Nucleic acid amplification test
NG: *Neisseria gonorrhoea*
NPV: Negative predictive value
PCR: Polymerase chain reaction
PI: Principal investigator
PIS: Patient infected status
PICO: Population intervention comparison outcome
PPV: Positive predictive value
PRISMA: Preferred reporting items for systematic review and meta-analyses
QUADAS-2: Quality assessment of diagnostic accuracy studies 2
RevMan: Review manager
STI: Sexually transmitted infections
SROC: Summary receiver operating characteristic
TN: True negative
TP: True positive
TV: *Trichomonas vaginalis*
WHO: World Health Organization
